# Transcriptome Sequencing Data Sets of Human Lung Epithelial Cells in the Course of Francisella tularensis Infection

**DOI:** 10.1128/MRA.00991-20

**Published:** 2020-10-15

**Authors:** Adi Bercovich-Kinori, Ma’ayan Israeli, Theodor Chitlaru, Inbar Cohen-Gihon, Ofir Israeli, Ofer Cohen

**Affiliations:** a Department of Biochemistry and Molecular Genetics, Israel Institute for Biological Research, Ness-Ziona, Israel; Indiana University, Bloomington

## Abstract

Francisella tularensis is a highly infectious intracellular bacterium representing the causative agent of tularemia, a severe disease which requires prompt antibacterial intervention for mitigating its potential high mortality. Inhaled bacteria interact with lung cells belonging to various subpopulations, including those of the epithelium. As of today, the host epithelial response to inhalational infection with F. tularensis is poorly understood. Here, we announce host transcriptome data sets which systematically address the human epithelial response to F. tularensis at different time points postinfection.

## Abstract (precis)

Francisella tularensis is a highly infectious intracellular bacterium representing the causative agent of tularemia, a severe disease which requires prompt antibacterial intervention for mitigating its potential high mortality. Inhaled bacteria interact with lung cells belonging to various subpopulations, including those of the epithelium.

## ANNOUNCEMENT

The facultative intracellular Gram-negative zoonotic pathogen Francisella tularensis is the causative agent of tularemia, a severe bacterial infectious disease of humans that is endemic in some parts of North America and Eurasia (1, 2). Due to its unusually high infectivity and the respiratory character of tularemia, F. tularensis has been classified as a category A select agent potentially associated with bioterrorism use (3–7). Previous *in vivo* studies have identified the epithelium cells as an important population affected in the course of F. tularensis infection (8, 9). However, little is known about the survival and replication of F. tularensis in these cells and about the creative strategies they use to maneuver their host cells. Here, we present transcriptome sequencing (RNA-seq) data of A549 human lung epithelial cells during F. tularensis infection.

Francisella tularensis subsp. *holarctica* strain LVS (ATCC 29684) expressing green fluorescent protein (GFP) was used for the infection of A549 (ATCC CCL-185) cells. Bacterial cultures were grown at 37°C to mid-log phase in TSBC (tryptic soy broth [Difco] supplemented with 0.1% cysteine [BD, France]). The bacteria were washed with phosphate-buffered saline (PBS) and resuspended in Dulbecco modified Eagle medium (DMEM) supplemented with 10% heat-inactivated fetal bovine serum. A549 cells were seeded 20 h prior to infection in 6-well microtiter plates at a density of 10^6^ cells per well. The cells were infected, in biological duplicates, with the overnight LVS culture to produce a multiplicity of infection of 20,000, centrifuged at 600 × *g* for 5 minutes, and incubated at 37°C for 0.5, 1, 3, 6, 12, and 24 hours. Following incubation, the 30-minute and 1-hour time point cultures were washed three times with PBS and subjected to RNA extraction using the RNeasy kit (Qiagen), while residual DNA was digested using RNase-free DNase (Qiagen). For subsequent time points, 1 hour postinfection, the medium was replaced with DMEM supplemented with 50 μg/ml gentamicin to kill extracellular bacteria. Thirty minutes later, cells were washed three times with PBS and left with DMEM supplemented with 10% FBS. At the indicated time points (3, 6, 12, and 24 hours postinfection), total RNA was similarly extracted.

RNA-seq was performed at the JP Sulzberger Columbia Genome Center (New York, NY). Libraries were generated using the Illumina TruSeq stranded mRNA kit according to the manufacturer’s instructions. Polyadenylated RNA enrichment was performed. Sequencing of the 100-bp paired-end reads was performed on the Illumina NovaSeq 6000 system. Pseudoalignment to a kallisto index created from transcriptomes (human GRCh38) was performed using kallisto (0.44.0). For RNA-seq quality control, we used fastQC v0.11.5, checked for per-base sequence quality, per-sequence quality scores, and adapter content. We verified that each sample reached at least 75% of the target read goal, and we checked for adequate sequence alignment percentages. An analysis of differentially expressed genes under various conditions was performed using the R package DESeq2 v1.18.1, with default parameters.

Sequencing yielded 19.6 million to 27 million reads per sample (Table 1), resulting in the identification of 21,066 transcripts. Differential expression along infection was demonstrated for 9,375 genes with more than 150 uniquely aligned reads of RNA.

**TABLE 1:** Summary of transcriptome samples submitted^*a*^

Sample description^*b*^	No. of reads	% mapped reads^*c*^	SRA accession no.	GEO accession no.
A549 mock_rep1	20,678,853	88.2	SAMN15775158	GSM4718274
A549 0.5 hpi_rep1	25,554,304	87.1	SAMN15775157	GSM4718275
A549 1 hpi_rep1	27,287,012	89.7	SAMN15775156	GSM4718276
A549 3 hpi_rep1	21,438,963	88	SAMN15775115	GSM4718277
A549 6 hpi_rep1	19,664,127	87.6	SAMN15775151	GSM4718278
A549 12 hpi_rep1	20,477,312	88.5	SAMN15775150	GSM4718279
A549 24 hpi_rep1	22,983,964	88	SAMN15775149	GSM4718280
A549 mock_rep2	21,168,131	88.5	SAMN15775148	GSM4718281
A549 0.5 hpi_rep2	20,860,054	87.4	SAMN15775145	GSM4718282
A549 1 hpi_rep2	22,230,500	89.2	SAMN15775144	GSM4718283
A549 3 hpi_rep2	20,751,390	86.3	SAMN15775143	GSM4718284
A549 6 hpi_rep2	25,684,370	85.6	SAMN15775142	GSM4718285
A549 12 hpi_rep2	23,009,615	87.5	SAMN15775141	GSM4718286
A549 24 hpi_rep2	25,669,488	87.4	SAMN15775140	GSM4718287

a The GEO accession number of the transcriptome series is GSE155970, and the SRA BioProject number is PRJNA656244. The GEO title of the project is “Transcriptome sequencing data sets for human epithelial cells response during Francisella tularensis infection.”

b Biologically duplicated samples are denoted as rep1 or rep2.

c Percentage of reads mapped to the reference genome (human GRCh38).

This study represents the first detailed transcriptome analysis of human epithelial response to Francisella tularensis infection and enables the identification of specific host factors involved in the pathophysiology of tularemia.

### Data availability.

The transcriptomic data have been deposited to the NCBI database, and their SRA and GEO accession numbers are provided in Table 1.
